# Enhanced prey capture skills in *Astyanax* cavefish larvae are independent from eye loss

**DOI:** 10.1186/2041-9139-5-35

**Published:** 2014-10-03

**Authors:** Luis Espinasa, Jonathan Bibliowicz, William R Jeffery, Sylvie Rétaux

**Affiliations:** School of Science, Marist College, 3399 North Rd, Poughkeepsie, NY 12601 USA; Equipe Développement Evolution du Cerveau Antérieur, UPR3294 N&D, CNRS, Institut Alfred Fessard, 91198 Gif-sur-Yvette, France; Department of Biology, University of Maryland, College Park, MA 02543 USA

**Keywords:** *Astyanax*, Lensectomy, Prey capture, Cavefish, Troglomorphy

## Abstract

**Background:**

Enhanced food-finding efficiency is an obvious adaptive response to cave environments. Here, we have compared the food-finding abilities of *Astyanax* surface fish and blind cavefish young larvae in their first month of life, in the dark.

**Results:**

Our results show that enhanced prey capture skills of cavefish are already in effect in fry soon after the yolk is depleted and the young larvae must find food for themselves. Moreover, using prey capture competition assays on surface fish fry with lensectomies, we showed that eye-dependent developmental processes are not the main determinant for enhanced prey capture skills. Finally, using F_2_ hybrid larvae resulting from crosses between surface fish and cavefish, we found that reduced eyes do not confer a selective advantage for prey capture by fry in the dark.

**Conclusion:**

We discuss these data with regards to our current developmental and genetic understanding of cavefish morphological and behavioral evolution.

## Background

A significant modification in feeding skills could be an evolutionary key to adapt to a new environment. Food scarcity and/or the lack of visual cues in caves can act as strong selective agents. An enhanced food-finding efficiency is an obvious adaptive response to environments that lack light and are often poor in food. For example, the cave crayfish *Orconectes inermis*[1], the cave salamander *Proteus anguineus*[2], and the spring cavefish *Chologaster Agassizi*[3] have a higher performance in prey detection and/or feeding success in the darkness when compared to closely related surface species (discussed in [4]).

The blind Mexican tetra *Astyanax mexicanus* is a model system in evolutionary developmental biology, which has provided an unprecedented understanding of the genetic and developmental controls of troglomorphic features. There are 30 known caves harboring *Astyanax* cavefish populations in México [5, 6] and their closely related surface-dwelling morph abound in nearby surface streams and throughout most of Mexico. *Astyanax* is well fitted for laboratory research as surface fish and cavefish are inter-fertile as well as suitable for experimental manipulations [7, 8]. In the literature it is often stated that the adult cave morph is more efficient in finding food in darkness. Specific modifications have been described to support this statement, such as a higher number of taste buds [9, 10], higher chemosensory capabilities [11, 12], an enhanced number of cranial neuromasts [13], modulation in early developmental signaling pathways influencing brain development and organization [14, 15], and a behaviorally more efficient posture with respect to the substrate when bottom feeding [16]. However, increased food finding efficiency is to our knowledge supported by only three controlled observations or experiments in which cavefish directly outcompeted surface fish for a limited amount of food.

- In the Hüppop [4] food-finding ability experiment, six surface and six Pachón cavefish aged at least 1.5 years (adults) were put in the dark to compete for single 10 mm^3^ pieces of beef-heart muscle, provided one at a time. About 80% of all food particles were found and eaten by cavefish.
- In the Yoshizawa et al. [17] prey capture competition assay, a pair of 2.5 to 4.0 cm long surface fish and Pachón cavefish were put in the dark and were provided with three drops of living *Artemia* larvae. Strikes at prey were recorded for 1 min with an infrared camcorder. About 70% of all strikes were performed by cavefish.
- In Wilkens and Hüppop [18], measurements of the population’s condition factor, that is, the relationship of body weight and body length as a measure of the nutritional state of a fish, were reported. In a few caves the cavefish live associated with surface fish, which sometimes are washed into the cave by flooding or by swimming upstream from springs. In caves where food is abundant, such as in the Chica cave where the guano of a large bat colony is available, surface fish have a comparable condition factor to the cavefish. On the contrary, in caves where food is scarce, such as the Río Subterráneo cave in the Micos area, surface fish washed into the cave look undernourished, have very low condition factor, and seem to be unable to compete with the local cave fish. The authors of the current paper have personally corroborated this observation and witnessed in multiple trips to this cave that in the first pool of the Río Subterráneo cave, surface fish are thin, and often appear to be dying or are already dead. In contrast, troglomorphic fish swimming in the same pool are well nourished.

A related feeding competition experiment was carried out by Sadoglu [19], but in this case the possibility of coupling between the selective value of the eye loss and other troglomorphic features was studied using the F_2_ progeny of a Pachón cavefish and surface fish cross to segregate the eyeless phenotype from other characters. For this experiment, 208 F_2_ larvae were put in dark or in light conditions and were given large amounts of *Artemia* larvae during the first month, complemented with dried food starting on week 2. After 3 months and 33% mortality, the surviving fish were classified according to eye morphology. Results showed that eye phenotypes (eye size) were statistically the same in both environments. Sadoglu concluded that ‘in mixed populations where interactions of phenotypes exist and where there is abundant food, eye type seems not to be selected: Survival value of each type is the same… we do not know what would be the result if the experiments were repeated under reduced food conditions’.

Eye degeneration in *Astyanax* is triggered by lens apoptosis [20, 21]. Using tissue transplantation, Yamamoto and Jeffery [21] reversed the loss of eyes in *Astyanax* cavefish. Surface fish lens vesicles were transplanted into cavefish embryonic optic cups, inducing the development of large eyes. Conversely, cavefish lens vesicles were transplanted into surface fish embryos and this resulted in adults with degenerate eyes. Likewise surface embryos on which lensectomy had been performed also resulted in adults with degenerated eyes. The physical presence of an eye has an effect on the developmental structure of the skull and the position of the suborbital bones [22], and the number of the small mandibular teeth [23]. But the most intriguing eye-dependent developmental effect is that the distance between the nasal and antorbital bones is enlarged when an eye is absent during development. Direct measurements of the size of the olfactory pits confirmed that a wider olfactory pit is present on eyeless cave fish and surface fish with a degenerate eye, and a narrower olfactory pit in eyed surface fish and cavefish with a restored eye. The width of the olfactory pit is modified by an average 12.9% due to the eye-dependent developmental processes [22]. An enlarged olfactory pit could result in an enhanced sense of smell, which could directly correlate with feeding skills of eyeless fish [12].

In recent years, much of the evolutionary developmental studies in *Astyanax* have been conducted in young fish. Results show that drastic changes with significant morphological and physiological outcomes occur within the first days after fertilization. The first objective of this paper is to describe a technique for assessing feeding skills after the yolk has just disappeared and the young larvae must find food for themselves. The second objective is to establish if the eye-dependent developmental processes by themselves are enough to account for the improved feeding skills in *Astyanax* cavefish larvae. The final objective is to replicate Sadoglu’s experiment of decoupling the selective value of the eye loss from other troglomorphic features by using F_2_ progeny, but under reduced food conditions that would foster high selective competition for prey capture skills.

## Methods

### Specimens

*Astyanax mexicanus* surface and Pachón cavefish were obtained from the Jeffery lab (University of Maryland, College Park, MD, USA) in 2004. Since then they have been maintained at the CNRS facility at Gif sur Yvette, France. Surface fish had initially been collected in San Solomon Spring, Balmorhea State Park, Texas, and the cavefish from Pachón cave, in Mexico. Spawning was induced as in [24]. The spawn of cave and surface fish was kept in 90 mm Petri dishes in an incubator at 23°C in ‘blue water’ (1 g.L^-1^ NaCl, 30 mg.L^-1^ KCl, 40 mg.L^-1^ CaCl_2_, 160 mg.L^-1^ MgSO_4_, and trace of methylene blue as anti-infectious agent) changed daily [8]. They were divided into four groups: cavefish in a 14:10 hour light/dark cycle, surface fish in a 14:10 hour light/dark cycle, surface fish under constant darkness, and surface fish which had undergone lensectomy under constant darkness. The two groups that were kept in constant darkness where derived from the same brood. After week one, all groups were fed daily with 2-day-old *Artemia* nauplii.

### Lens removal

Lens removal was conducted bilaterally in surface fish at 1 to 2 days post fertilization following the basic procedure outlined by [25], with some alterations. The spawned eggs and pre- and post-operation hatched larvae were incubated in blue water. Specimens were incubated in anesthetizing solution (0.1 mg/mL MS222 Ethyl-3-aminobenzoate methanesulfonic acid salt plus 0.1 mg/mL NaHCO_3_) for 2 min before operation. They were then mounted in 2% low melting agarose in anesthetizing solution and lensectomies were performed under a dissection microscope. For the micromanipulations, instead of using tungsten needles held by hand, microinjection needles were made from glass capillaries with a Narishige’s PC-10 Dual-Stage Glass Micropipette Puller and attached to a manual micromanipulator (Type MM33 Rechts; Märzhäuser, Wetzlar, Germany). Bilateral lensectomies using microinjection needles made from glass capillaries attached to a manual micromanipulator were extremely successful when compared to previous results using tungsten needles held by hand (Hélène Hinaux, personal communication), with postoperative survival nearly 100%. In the brood used for this experiment, no single specimen died during the operation or throughout the following 4 weeks of incubation. All treated specimens developed normally and had equivalent sizes to their untreated siblings. The success of the lensectomies was evident by the specimens’ significantly smaller eyes when compared to untreated specimens of the same brood at the time of the test. After operation, specimens were then rinsed in blue water, released from the agar mounting medium and returned to the incubator where they were kept in Petri dishes at 23°C. Before and after lensectomies, fish were kept in the dark in boxes. As a control, fry from the same brood were kept also in the dark in the same incubator side by side with the experimental specimens, so as to have the same genetic background and to experience the same environmental conditions, with the exception of the lensectomy.

### Prey capture competition assay

**Figure 1:**
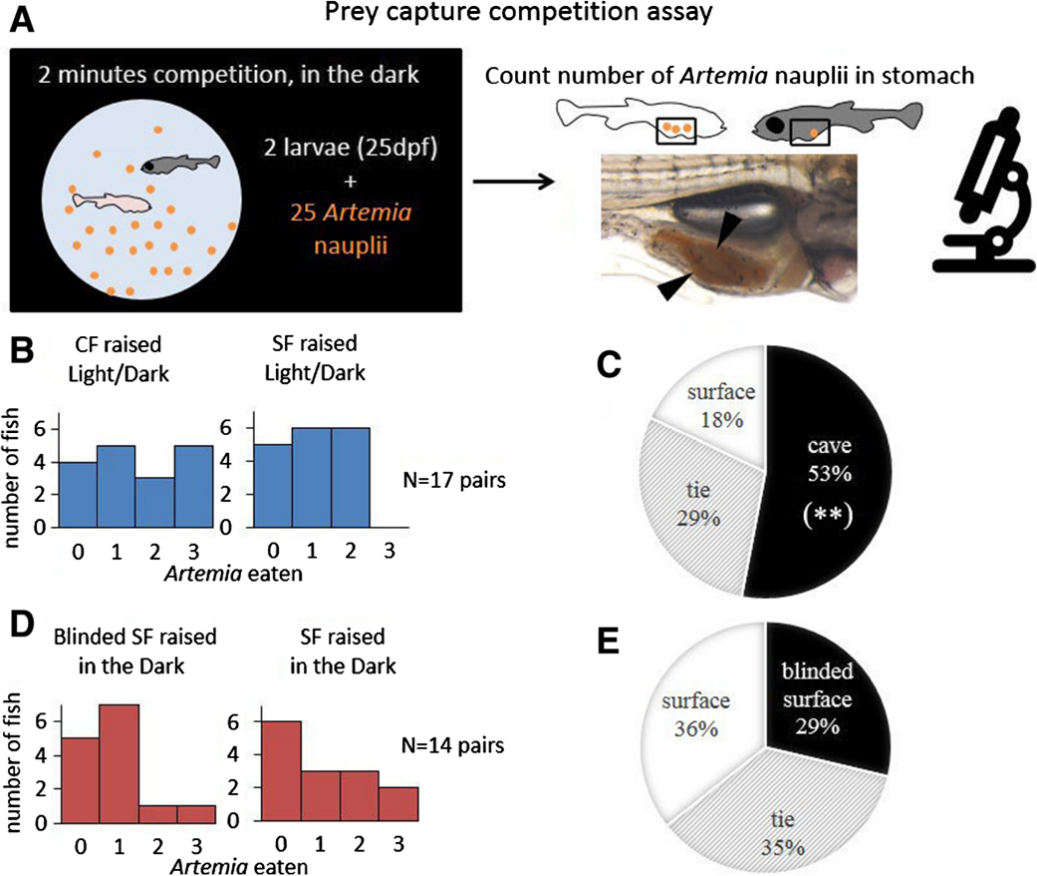
**Prey competition in the dark with pairs of 4 week post-fertilization fish larvae. (A)** Diagram of the experimental design. **(B,** **D)** Distribution histogram showing the number of *Artemia* nauplii eaten by each type of fish during the paired competitions. In **(B)**, *P* = 0.076 and in **(C)** *P* = 0.7253 for paired matches comparisons (Wilcoxon paired test). **(C,** **E)** Pie charts showing the distribution of ‘winner’ fish, that is, the fish type showing the highest numbers of *Artemia* larvae in their stomach. Cave fish globally outcompeted surface fish in the prey capture competition (**C**, *P* = 0.0097**; Fisher’s exact test), while surface fish with lensectomy performed equally well to surface fish raised in darkness (**E**, *P* = 0.7688; Fisher’s exact test).

Specimens at 25 day post fertilization were starved for 36 h in the dark. The two competing individuals were then transferred into one of the wells of a 6-well non-treated culture plate (Costar) with 4 mL of water. Specimens were acclimated for 2 h in the dark, then fed with an estimated 25 *Artemia* nauplii (2 days old) in 1 mL of water and the two fish were allowed to eat for 2 min in the dark, after which 1 mL of 20X anesthetizing solution was added to terminate feeding behavior. Individual fish were immediately observed under a dissection microscope and *Artemia* larva in the stomach were counted. For effective visualization, light from above was used to reflect the red contrasting color of *Artemia* nauplii which were easily counted using their shape, size, eyes, and color (Figure 1A).

Two tests were performed. In the first, the pair of individuals were a cavefish and a surface fish, both raised under light conditions. Seventeen replicates were performed. In the second test, the pair of individuals were a surface fish on which lensectomy had been performed and a normal surface fish, both derived from the same brood and both raised in the dark. Fourteen replicates were performed. In all tests all individuals were used in a single match. The same specimen was never used in a second match. For Figure 1B and D we have applied a Wilcoxon paired test (because the two fish are in competition and therefore interact). For Figure 1C and E a Fisher’s exact test was used (because the sample size is moderate). Statistics were performed with R.

### F_2_ competition assay

The brood from a tank with F_1_ progeny derived from Pachón cavefish and surface fish was incubated in the darkness for 6 days in standard fish tank water, as in Sadoglu [20]. Afterwards, 200 of the F_2_s were transferred to 14:10 hour light/dark conditions and another 200 were kept under continuous darkness. Food was then provided exclusively every third day, and only about 1,000 *Artemia* per time. This is equivalent to about five *Artemia* per fish every third day. Specimens were sacrificed and fixed after the fourth feeding event, when fish were 17 days post fertilization. Eyes were measured under a dissection microscope to the nearest 0.001 mm. A Wilcoxon test (with R) was used to determine if eye size was different in the F_2_ progeny kept in the illuminated or dark conditions.

## Results

### Comparing food finding abilities of cavefish and surface fish larvae in the dark

In the first test, 25-day-old cavefish and surface fish raised under light/dark conditions were paired to compete for *Artemia* nauplii in the dark (Figure 1A). On average, cavefish captured 1.59 *Artemia* in each paired match, while the surface specimens captured 1.05 *Artemia*. It is worthwhile mentioning that in the allotted 2 min for prey capture, on five occasions the cavefish was able to eat three *Artemia.* The maximum number captured by surface fish in this test was two (Figure 1B). In the 17 matches performed, cavefish were more successful in capturing prey than their surface counterparts (Figure 1C). In nine (52.9%) competitions the cavefish had more *Artemia* larvae in their stomachs than the surface fish. On five (29.4%) occasions they both had the same amount and in only three (17.6%) matches did the surface fish capture more prey than the cavefish (Figure 1C; *P* = 0.0097**; Fisher’s exact test). This shows that, already at larval stages, cavefish have better food finding abilities than surface fish in the dark.

### Do eye-dependent developmental processes explain differences in food-finding abilities in the dark?

On the second test, normal surface fish and lensectomied surface fish from the same brood, both raised under constant dark condition, were paired. In the 14 matches performed, both groups were equally successful in capturing prey. On average, blinded surface fish captured 0.7 *Artemia* while surface fish raised in the dark captured 0.88 *Artemia*. In four (28.6%) competitions the surface fish with lensectomy had more *Artemia* larvae in their stomachs than the control surface fish. On five (35.7%) occasions they both had the same amount and on five (35.7%) matches the normal surface fish captured more prey than the surface fish with lensectomy. In sum, the two types of surface fish appeared equally efficient to find *Artemia* (Figure 1E; *P* = 0.7688; Fisher’s exact test). This suggests that the eye-dependent developmental processes in *Astyanax* are not a major factor in promoting the enhanced prey capturing skills, at least under current testing conditions.

### Does the loss of eyes confer a selective advantage for finding food in the dark?

**Figure 2:**
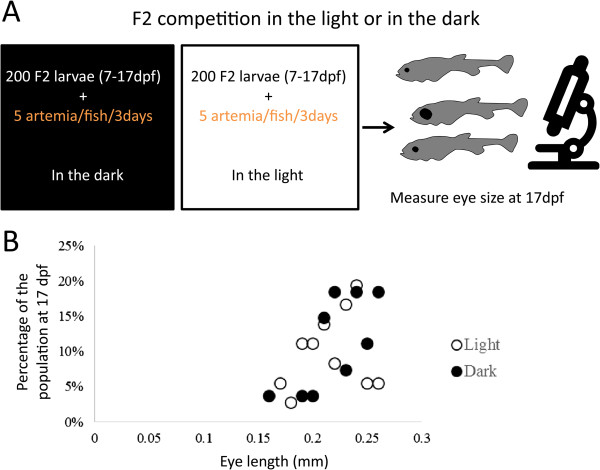
**Food competition experiment between F**_**2**_ **fish larvae, in the light or in the dark. (A)** Diagram of the experimental design. **(B)** Plot showing the distribution of eye sizes in survivors (only 15% of the 200 initial larvae) after 10 days of competition for very limited amounts of food (about five *Artemia* larvae per fish every third day). The two groups of F_2_s have similar eye sizes (*P* = 0.388; Wilcoxon test).

To further assess the eye-independence of food finding abilities in *Astyanax*, and to assess the selective value of eye loss in the increased food finding abilities of cavefish, we turned to genetics. We took advantage of the fact that surface fish and cavefish can breed and generate F_1_ and F_2_ progenies. Two groups of F_2_ larvae originating from the same brood that had survived after competition for reduced food under illuminated or dark conditions had their eyes measured (Figure 2A). Average eye size of the F_2_ survivors kept in illuminated conditions was 0.219 mm +/- 0.025 SD while the average eye size of those who survived in the dark was 0.230 mm +/-0.025 SD. The two groups of F_2_s did not have significantly differently sized eyes (*P* = 0.388; Wilcoxon test) (Figure 2B), indicating that a dark environment during embryonic and larval stages does not strongly select for reduced eye size, at least to a level detectable under current testing conditions.

This corroborates Sadoglu’s [19] results, where reduced eye size was not selected for in the dark. Selective conditions were more stringent in our study than in Sadoglu’s experiment. Food amounts were much reduced and at the end of our study (17 days post fertilization), mortality was 85%, which is much higher than the 33% mortality after 3 months in Sadoglu’s experiment. Despite this, our study also failed to find a selective advantage for smaller eyes when competing for limited amounts of prey in the dark. This suggests that pleiotropic effects of the factors involved in the genetic control of eye size are not major factors in promoting the enhanced prey capturing skills of cavefish, at least under current testing conditions with fish larvae.

## Discussion

Few studies have directly tested if cavefish outcompete surface fish for a limited amount of food and all have been performed in adult fish [4, 16, 17]. Our results corroborate results from these studies but most importantly have shown that the enhanced feeding skills of cavefish are already in effect in fry soon after the yolk is depleted and the young larvae must find food for themselves. When a pair of 4-week-old cavefish and surface fish compete for *Artemia* larvae over a 2-min period, cavefish outcompete surface fish three times more often than the surface fish outcompete cavefish.

The second part of this study sought to uncover whether the enhanced prey capture skills displayed by cavefish fry depend on eye loss. Sonic Hedgehog (Shh) gene expression is expanded along the anterior embryonic midline in cavefish. Shh hyper-signaling indirectly (by an unknown molecular pathway) results in lens apoptosis and arrested eye growth and development [14]. The degeneration of the eye modifies craniofacial development, which ultimately leads to differences in the cavefish skull and wider olfactory pits in adults [22]. In addition to negative effects on eye development, Shh hypersignaling is related to the evolution of several constructive traits that could modulate feeding skills independently of the eye. These include increased jaw size and taste bud numbers [9, 10] and differences in ventral forebrain regions [26, 27]. In addition, Fibroblast Growth Factor 8 (Fgf8) signaling is heterochronic in cave and surface embryos, with an indirect impact on the size of the retina [15], and this signaling molecule is also prone to have important morphogenetic effects.

The appeal of lensectomy procedures is that they allow decoupling of eye-dependent developmental processes that take place after 1 to 2 days post fertilization (that is, the stage when the lens is removed) from the other pleiotropic effects of signaling molecules (such as Shh and Fgf8) on the differences in prey capture skills between cavefish and surface fish. Specimens with the genetic background of a surface fish (including Shh/Fgf8 controlled developmental structures), but with degenerated eyes (including eye-dependent developmental structures) can be compared to normal surface fish. Furthermore, by raising them both in continuous darkness, phenotypic plasticity is controlled.

Our results suggest that the eye-dependent developmental effects are not responsible for the enhanced prey capture skills assessed in our experimental setup and which are already evident in cavefish at this stage. Surface fish that had undergone lensectomies and had conspicuously smaller eyes did not out-compete normal surface fish that had been raised in the dark.

The aim of the last part of the study was to assess the selective value of the eye in F_2_ progeny under strongly selective conditions of scarce prey. If the genes regulating eye size were pleiotropic and also controlled the feeding behavior of fry and strong selection provided an advantage when limited prey is available, it would have been expected that in the F_2_ test, small eyed fish should have shown greater survivorship. This was not the case, neither in our test on fish larvae with limited food nor in Sadoglu’s 3-month-old fish with sufficient food [19]. This implies that eye size causative and dependent processes did not have a strong selective value for prey capture in young *Astyanax* under the conditions tested. Much caution should be used not to misconstrue and overvalue this conclusion. Previous quantitative genetic studies have established that eye degeneration in *Astyanax* is a complex trait caused by numerous mutations of small effect. Quantitative trait loci (QTL) mapping has identified 8 to 12 QTL for eye size [11], and QTL polarities actually suggest that eye regression occurred through selection [28]. With the sample size available in these studies (15% survival from a starting population of 200 and 66% survival from two starting populations of 208 each) and the confounding factor of multifactorial genetic determinants of eye size, small selective effects for eye size could go undetected. Nonetheless, these studies suggest that eye size is not the strongest and main determinant of cavefish enhanced prey capture skills. Finally, the pleiotropic effects of reducing eye size may still be selected for, but in older fish or for other skills different than prey capture of small crustaceans, such as olfaction.

Results from our experiment make sense *a posteriori*. While enlarged olfactory pits, taste buds and smell may be beneficial for detecting the odors of food, they may not be the most important factor in capturing a moving live prey. We are well aware that complex characters such as feeding skills must be modulated by a large set of multifactorial causative agents. Furthermore, feeding success may rely on different resources depending on the type of food available. For example, cavefish may need a completely different set of sensory and behavioral devices to eat a guano dropping that happened to be far away in a pool, from those needed to capture a small crustacean that whirls fast past its face. By force, our experimental setup could only assess causative agents for the skills required to prey on *Artemia* nauplii, at short distances in a limited amount of time.

Small invertebrates such as copepods disturb the water at 30 to 40 Hz when swimming [29], which is in the detection range of the superficial neuromasts in the lateral line system [17]. Vibration attraction behavior (VAB) is the ability of fish to swim toward the source of a water disturbance and has been shown to be advantageous for *Artemia* capture in the dark by adult cavefish [17]. The cupulae (hair cell stereocilia covered by a gelatinous case) of cavefish superficial neuromasts are about 300 μm in length compared to about 42 μm in surface fish [30]. Neuromasts within the eye orbit and in the suborbital region are larger and consequently about twice as sensitive in young adult cavefish as in surface fish [31]. VAB is typically seen in cavefish, but rarely in surface fish. While VAB is not statistically evident before cavefish reach 3 months of age [17], it is possible that some of the physiological bases behind the behavior are starting to be active in young fish. Yoshizawa et al. [13] showed that experimental induction of eye regression in surface fish via Shh overexpression was insufficient to promote the appearance of VAB or eye orbit superficial neuromasts. Yoshizawa et al.’s results and ours are congruent because eyeless surface fish, brought about by either Shh overexpression or by lensectomy, would both lack the enhanced VAB or superficial neuromasts activity of a cavefish, and would not be efficient at prey capture.

## Conclusions

Our results suggest that at a young stage when the yolk has been depleted and the young larvae must find food for themselves, *Astyanax* cavefish already have enhanced skills for prey capture. This modification in feeding skills in fry is probably essential for the survival within the cave environment. These skills are primarily modulated by processes that are independent of eye loss. This is congruent with Shh-independent processes, such as the enhancement of superficial neuromasts activity, for example. Other eye-independent options are feasible, such as enhancement of mechanosensors, chemical sensors (nasal epithelium and tastebuds), or performance of the brain, to name a few.

Eye-dependent developmental processes may still be involved in the enhancement of feeding skills but not assessed in our experimental setup. Cavefish probably feed on a variety of stationary and moving items in cave pools. Stationary objects located at the bottom of cave pools, such as particles of bat guano, could be more efficiently detected using olfactory cues and an enlarged olfactory pit may still prove beneficial. Future studies targeting smell detection in surface fish that have undergone lensectomy, or fish treated with gentamicin, a neuromast inhibitor, may be able to further resolve this interesting issue.
